# Gut-Derived Peptide Hormone Analogues and Potential Treatment of Bone Disorders in Obesity and Diabetes Mellitus

**DOI:** 10.1177/11795514241238059

**Published:** 2024-03-13

**Authors:** Asif Ali, Peter R Flatt, Nigel Irwin

**Affiliations:** Diabetes Research Centre, Biomedical Sciences Research Institute, Ulster University, Coleraine, Northern Ireland, UK

**Keywords:** Diabetes, obesity, bone, incretin, peptide, analogue

## Abstract

Obesity and diabetes mellitus are prevalent metabolic disorders that have a detrimental impact on overall health. In this regard, there is now a clear link between these metabolic disorders and compromised bone health. Interestingly, both obesity and diabetes lead to elevated risk of bone fracture which is independent of effects on bone mineral density (BMD). In this regard, gastrointestinal (GIT)-derived peptide hormones and their related long-acting analogues, some of which are already clinically approved for diabetes and/or obesity, also seem to possess positive effects on bone remodelling and microarchitecture to reduce bone fracture risk. Specifically, the incretin peptides, glucagon-like peptide-1 (GLP-1) and glucose-dependent insulinotropic polypeptide (GIP), as well as glucagon-like peptide-2 (GLP-2), exert key direct and/or indirect benefits on bone metabolism. This review aims to provide an initial appraisal of the relationship between obesity, diabetes and bone, with a focus on the positive impact of these GIT-derived peptide hormones for bone health in obesity/diabetes. Brief discussion of related peptides such as parathyroid hormone, leptin, calcitonin and growth hormone is also included. Taken together, drugs engineered to promote GIP, GLP-1 and GLP-2 receptor signalling may have potential to offer therapeutic promise for improving bone health in obesity and diabetes.

## Plain Language Summary

Impact of peptides from the gut on bone health in obesity and diabetes mellitus

Obesity and related type 2 diabetes (T2D) are prevalent diseases. Unfortunately, there is now a clear link between obesity and related T2D and poor bone health, leading to increased bone fracture risk. However, we know that peptides derived from the gut following a meal can possess positive effects on bone health and reduce bone fracture risk. These peptides are called glucagon-like peptide-1 (GLP-1), glucagon-like peptide-2 (GLP-2) and glucose-dependent insulinotropic polypeptide (GIP). Moreover, some of these peptides, GLP-1 and GIP, are already being used to treat obesity and T2D, whilst GLP-2 is used to treat people with short bowel syndrome. In other words, drugs that mimic the action of GLP-1, GLP-2 and GIP are available for human use. This current review article aims to provide an initial appraisal of the relationship between obesity, diabetes and bone health, with a focus on the positive impact of peptide hormones like GLP-1, GLP-2 and GIP for bone health in obesity/diabetes. The take home message is that drugs engineered to promote GIP, GLP-1 and GLP-2 action may have potential to offer therapeutic promise for improving bone health in obesity and diabetes.

## Introduction

Obesity and type 2 diabetes mellitus (T2DM) are related diseases that have adverse effects on various physiological systems within the body.^1^ On a global scale, the prevalence of T2DM has increased dramatically in recent years, with over 1 in 10 adults now currently living with the condition.^2^ Since release of the International Diabetes Federation (IDF) data in 2000, the estimated occurrence of diabetes in individuals aged 20 to 79 has more than doubled, rising steadily from approximately 151 million in 2000 (4.6% of the world’s population) to 537 million (10.5% of the world’s population) in 2021. If appropriate measures are not taken to address this issue, projections indicate that this number will surge to an alarming 783 million individuals by 2045.^3^ In accord with this, the prevalence of obesity has more than tripled globally since 2016,^2^ and it is anticipated that by 2030 one billion people will be clinically obese.^4^

The increase in obesity and T2DM is partly related to a progressively ageing population, adoption of a more sedentary lifestyle and consumption of high energy processed foods.^5^ In keeping with this, the prevalence of osteoporosis and fragility bone fractures is also rising.^6^ More notably, obesity and diabetes independently increase bone fracture risk, linked to impairments in bone turnover and a detrimental alteration of bone microarchitecture.^7,8^ Consequently, there is a need to better understand how obesity and T2DM specifically impact bone health, with a view towards developing tailored treatments to address this unique challenge.

## Impact of Diabetes on Bone Health

**Figure 1.**
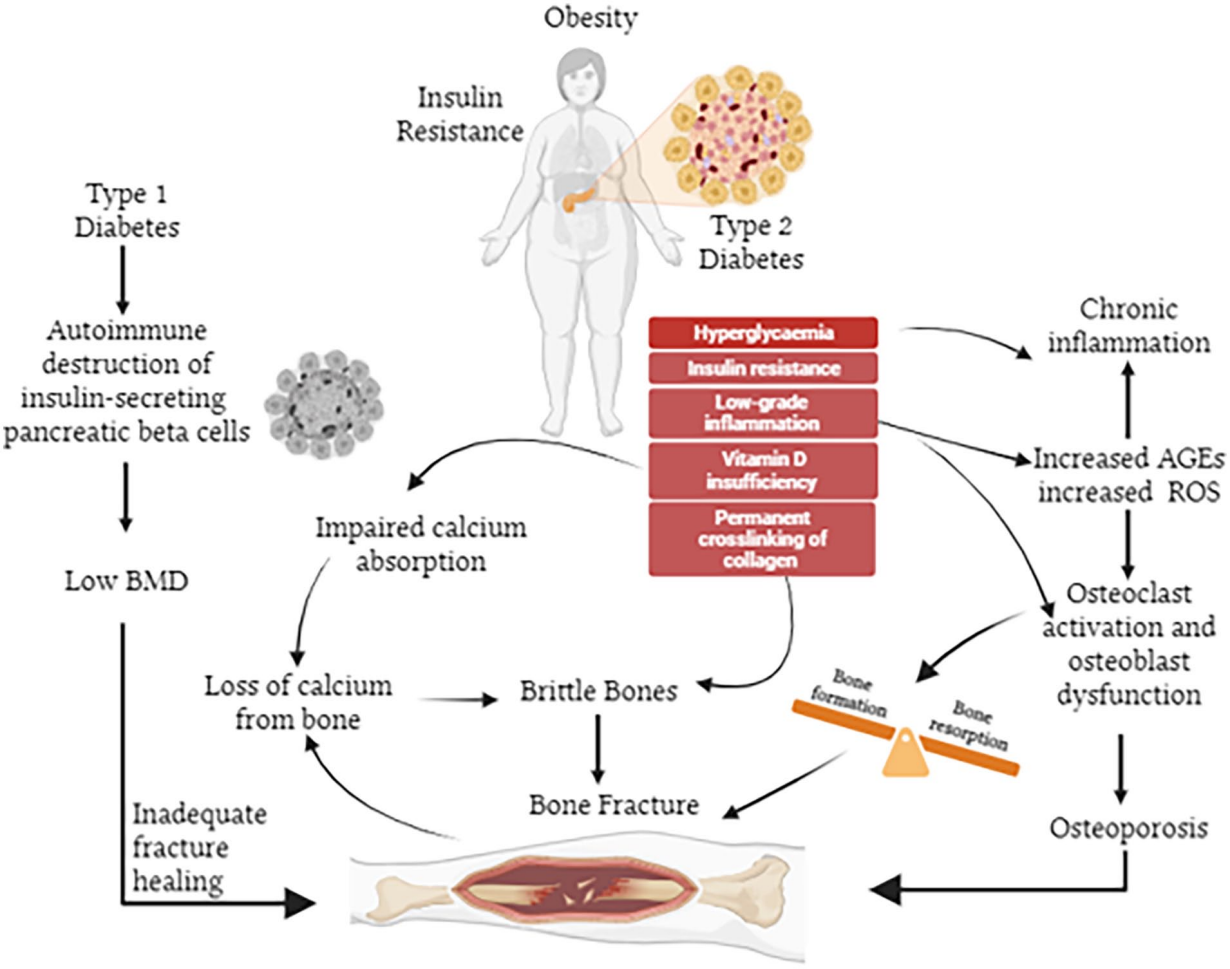
The complex interplay between diabetes, obesity, and bone health. T2DM is associated with a pro-inflammatory state induced by hyperglycaemia and obesity, resulting in insulin resistance. Several factors contribute to the deleterious effects of diabetes on bone health, including increased levels of AGEs and ROS. These factors play increase osteoclast activation and promote osteoblast dysfunction, ultimately culminating in bone disorders and increased fracture risk.

Osteoclasts and osteoblasts are key cells involved in the well-described bone remodelling process, which mediate bone resorption and subsequent bone formation, respectively.^9^ However, the equilibrium between bone development and bone resorption is disrupted in obesity and T2DM^10^ (Figure 1). As such, all forms of diabetes are associated with prolonged hyperglycaemia, that is known to negatively impact bone.^11^ In keeping with this, receptor activator of nuclear factor-κB ligand (RANKL), that simulates mature osteoclasts to resorb bone,^12^ also directly affects pancreatic beta-cell function and overall glucose metabolism but has impaired function in obesity/diabetes.^13^ Additionally, the RANKL monoclonal antibody used to treat osteoporosis, namely denosumab (Dmab), promotes pancreatic beta-cell proliferation^14^ and has recently been suggested to positively regulate glucose homoeostasis in patients with T2DM.^15^ However, benefits of Dmab on fasting glucose in T2DM were not superior to ibandronate therapy.^15^ In accordance with this, other studies indicate that RANKL increases hepatic and muscular insulin resistance and upregulates negative glycaemic regulators such as the adipokine resistin and the incretin-degrading enzyme dipeptidyl peptidase-4 (DPP-4).^13^ Hyperglycaemia can also trigger a rise in TNF-α and IL-6 expression, with these cytokines being linked to osteoclast development and activation,^16,17^ that ultimately promotes bone loss. Furthermore, a prolonged hyperglycaemic environment exerts a negative impact on osteoblast metabolism and maturation.^18^ Increased generation of advanced glycation end-products (AGEs) may also be involved in the bone defects of diabetes (Figure 1), with AGE-induced upregulation of sclerostin, an inhibitor of osteoblast function,^19^ disrupting the delicate balance between bone formation and resorption.^20^

Importantly, diabetes also negatively impacts bone micro-architecture at a structural level, and people living with diabetes have been found to have decreased trabecular bone connectivity and cortical bone thickness.^21^ Such structural changes render the bone more susceptible to fragility fracture and also impair the healing process.^22^ In agreement with this, osteocyte morphology and osteocyte network topology have been shown to be impaired in insulin-resistant mice fed a high-fat diet,^23^ whilst bone strength and cortical microstructure are impaired in insulin-deficient mice administered the specific pancreatic beta-cell toxin, streptozotocin (STZ).^24^ A brief overview of the influence of the 2 major forms of diabetes, namely type 1 diabetes mellitus (T1DM) and T2DM, on bone health is provided below. However, it should be noted that there is still an incomplete understanding of the complex mechanisms related to the negative impact of diabetes on bone microarchitecture (Figure 1).

### T1DM and bone health

T1DM is characterised by the autoimmune destruction of insulin-secreting pancreatic beta-cells, leading to insulin deficiency with obvious negative effects on bone health.^25-27^ Consequently, low BMD, increased risk of fractures and inadequate fracture healing are all observed in T1DM,^25,26^ with the disease being linked to early-onset osteopenia or osteoporosis.^27^ Indeed, a long-term observational study, known as the Epidemiology of Diabetes Interventions and Complications study (EDIC), following 1058 participants originally enrolled in the Diabetes Control and Complications Trial (DCCT), reveals that poor glycaemic control and AGE accumulation represent independent risk factors for lower hip BMD in older adults with T1DM.^28^ Furthermore, osteoblasts express the insulin receptor, where physiological insulin levels have been shown to enhance production of bone anabolic markers and collagen synthesis.^29-31^ Moreover, insulin triggers bone cells to synthesise and activate osteocalcin, an endocrine hormone that not only helps to coordinate appropriate bone formation, but also positively regulates glucose metabolism.^31^ Consequently, mice lacking the insulin receptor in osteoblasts have low levels of circulating osteocalcin and limited bone growth.^32^ Underlying mechanisms for disrupted bone physiology in T1DM include elevated levels of pro-inflammatory cytokines such as TNF-α, IFN-α, IL-6, IL-17 and IL-21, also thought to be involved in the onset and progression of T1DM,^33^ that ultimately contribute to bone loss by encouraging osteoclast differentiation and/or suppressing osteoblast development and function.^34^

### Obesity, T2DM and bone health

Insulin resistance, a prominent characteristic of obesity and T2DM, exerts a significant negative effect on skeletal integrity through the impairment of osteoblast activity and augmentation of bone resorption, mediated in part by relative insulin deficiency.^22^ In addition, obesity per se exerts a substantial influence on bone health, primarily by elevated mechanical stress with weight-bearing joints such as the knees and hips being particularly affected.^35^ Although sarcopenia, an age-related progressive loss of muscle mass and strength, can be difficult to define,^36^ it has been shown compromise bone health in obesity. In that regard, interventions to improve muscle mass may help lower fracture risk in sarcopenic obesity.^37^ Moreover, since adipocytes and osteoblasts are derived from the same multipotent mesenchymal stem cell, obesity may favour adipocyte over osteoblast differentiation.^38^ Obesity is also linked to a persistent state of low-grade inflammation and increased concentrations of specific cytokines such as IL-6 and TNFα, that can exert detrimental effects on bone health such as increasing bone resorption and impeding bone formation.^34,39^ Furthermore, individuals with obesity are more prone to reduced circulating levels of vitamin D, due to sequestration of the fat-soluble vitamin within adipose tissue.^40^ Accordingly, vitamin D deficiency contributes to a decline in BMD and augments susceptibility to bone fractures.^41^ Mechanistically, vitamin D insufficiency causes impaired calcium absorption, which eventually results in the release of calcium from bones in a bid to maintain normal circulating concentrations of the ion, compromising integrity of the bone.^42^ Furthermore, the bone remodelling process is modulated by adipokines including leptin, adiponectin and resistin,^43,44^ and circulating levels of these adipose-derived hormones are disrupted in obesity.^45^ Interestingly, leptin receptors are present on bone cells and contribute to the growth and mineralisation of osteoblasts,^46^ as well as inhibiting osteoclast activity,^47^ which may partly explain the unfavourable bone phenotype observed in obese-diabetic *db/db* mice.^48^ Finally, development of AGEs in obesity and diabetes leads to permanent crosslinking of collagen fibres and impairment of organic bone matrix quality, making the bone more brittle and prone to fracture^49,50^ (Figure 1). Moreover, osteoblast apoptosis is triggered by extracellular AGEs,^51^ linked to the downregulation of expression of genes necessary for osteoblast development, such as osterix and Runx2.^52,53^ In addition to this, microvascular disease is a well-defined complication of T2DM and has been shown to impair skeletal integrity and lead to increased bone fragility, primarily by detrimentally affecting osteocyte function.^54^

## The Gastrointestinal Tract and Bone

**Figure 2.**
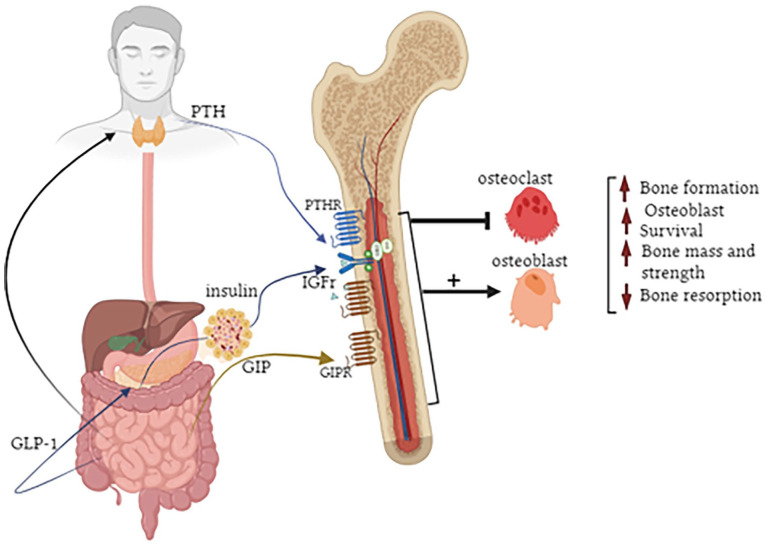
Positive impact of GIP, GLP-1 and GLP-2 on bone metabolism. GIP exerts direct benefits through activation of receptors present on bone. The positive bone effects of GLP-1 and GLP-2 may be mediated indirectly through promotion of insulin and PTH secretion, respectively. Together these GIT-derived peptides increase osteoblast survival and decrease bone resorption leading to improvements of bone formation as well as overall bone mass and strength.

As discussed above, several endocrine factors are known to regulate the bone remodelling process.^55^ Of particular interest in this regard, and with key relevance to obesity and diabetes, are hormones secreted from the gastrointestinal tract (GIT). As such, a role for GIT derived peptide hormones in the maintenance of bone integrity and strength has now largely been confirmed.^56^ In this respect, the incretin hormones, namely glucose-dependent insulinotropic polypeptide (GIP) and glucagon-like peptide-1 (GLP-1), have received much attention to date. GLP-1 mimetics are approved for the treatment of both obesity and T2DM,^57-59^ whilst a dual-acting GLP-1/GIP receptor co-agonist, namely tirzepatide, has also recently gained clinical approved for T2DM^60^ and obesity.^61^ Both GIP and GLP-1 are considered to either directly or indirectly affect the skeleton,^62^ with their activity and/or secretion detrimentally altered in obesity-diabetes, that might contribute to alterations of bone quality in this setting. Beyond this, the sister hormone of GLP-1, namely glucagon-like peptide-2 (GLP-2), now utilised in a form resistant to DPP-4 degradation for the treatment of short bowel syndrome,^63^ also exerts well established effects on bone metabolism.^64^ The following sections will highlight the role of GIP, GLP-1 and GLP-2 in bone biology (Figure 2), with specific reference to potential application for the treatment of bone disorders observed in obesity and diabetes. Given the wealth of information covered within these areas, summary tables that present the main findings from rodent and human studies have been generated (Tables 1 and 2). Although data derived from the clinic is encouraging (Table 2) there are limitations in terms of overall interpretation, since bone composition and material properties are unable to be fully investigated. Thus, positive observations with GIP, GLP-1 and GLP-2 receptor agonists on bone in rodents still need to be fully translated to the human setting.

**Table 1.** Overview of effects of gut-derived peptide analogues on bone health in animal models of diabetes and/or obesity and rodent cell lines.

Peptide name	Model	Key findings	Reference
Exenatide	*OVX rats*	Promotes osteoblast activation, enhances bone strength, inhibits bone resorption, and prevents deterioration of trabecular microarchitecture	Ma et al, 2013^105^
Liraglutide	*STZ-induced diabetes and/or OVX-rats/mice*	Reduces osteoclast numbers and protects against femoral bone mineral loss and deterioration of bone microarchitecture. Increased serum osteocalcin and decreased bone resorption markers.	Wen et al, 2018^106^ & Mansur et al, 2015^24^
(D-Ala^2^)GIP	*STZ-diabetic mice*, *Bone marrow cells*, *Raw 264.7 cells*	Increased bone quality and effectively halted degradation of bone matrix to improve bone fracture resistance. Reduced osteoclast formation and decline in bone resorption.	Mansur et al, 2019^112^ & Mabilleau et al, 2016^23^
N-AcGIP	*Bone marrow cell*, *MC3T3 cells*	Reduction in osteoclast count and CTX levels. Improved tissue material properties of bone. Increased alkaline phosphatase activity.	Mabilleau et al, 2016^23^
GL-0001 and GL-007 (GIP/GLP-2 co-agonists)	*OVX mice*, *Raw 264.7 cells*	Reduced osteoclast resorption. Preservation of trabecular bone mass. Enhanced collagen cross-linking. Improved fracture resistance	Gobron et al, 2023^126^
Lipidated long-acting GLP-2R agonists	*COS-7 cell line, transgenic mice and rats*	Trophic effects on bones. Lowered bone resorption CTX levels.	Gadgaard et al, 2023^85^
(D-Ala^2^)GIP-Oxm (GIP/GLP-1/glucagon triple agonist)	*db/db mice*	Improved bone strength following 3 weeks treatment	Mansur et al, 2016^11^
HM15211 (GIP/GLP-1/glucagon triple agonist)	*OVX rats*, *SaOS2 cell*	Protects against bone loss. Type 1 collagen-α1, -α2, and carboxylated osteocalcin expression significantly increased.	Kim et al, 2017^127^

**Table 2.** Overview of effects of gut-derived peptide analogues on bone health in humans with diabetes and/or obesity and human cell lines.

Peptide name	Model	Key findings	Reference
Exenatide	*T2DM*	Increase BMD and reduce fracture risk. Improved trabecular bone mass and architecture by increasing bone formation rate.	Cai et al, 2021^110^
Liraglutide	*Obesity/T2DM*	Reduction in the number of osteoclasts. Augmentation of bone formation, safeguarding against reduction of mineral content and bone structural integrity.	Xie et al, 2021^90^
Dulaglutide	*T2DM*, *SW1353 chondrocytes*	Increase BMD and reduce fracture risk. Diminishing ROS levels and expression levels of multiple pro-inflammatory cytokines and chemokines associated with osteoarthritis.	Cai et al, 2021^110^ & Li et al, 2020^154^
Lixisenatide	*Primary human chondrocytes*	Reduced the breakdown of type II collagen. Reduced IL-6 and TNF-α expression.	Li et al, 2019^107^
Various long-acting GLP-1 mimetics	*Obesity*	Enhanced bone growth and prevented bone loss.	Iepsen et al, 2015^108^
(D-Ala^2^) GIP	*Human peripheral blood mononuclear cells*	Directly inhibited osteoclast formation and resorption. Reduction in CTX levels.	Mabilleau et al, 2016^23^
N-AcGIP	*Human peripheral blood mononuclear cells*	Reduced osteoclast formation and resorption. Reduction in osteoclast count and CTX levels.	Mabilleau et al, 2016^23^

### GLP-2 and related analogues

Multiple peptides with overlapping functions that support the absorption and assimilation of nutrients are encoded by the proglucagon gene.^65,66^ Of these, GLP-2 is a 33 amino acid polypeptide secreted gut L-cells that was initially reported to exert an intestinal growth-promoting action.^67^ Similar to other endogenous regulatory peptides, GLP-2 has a short biological half-life and is readily broken down in the circulation by the enzyme DPP-4, to yield and inactive peptide metabolite.^68^ However, the half-life of GLP-2 can be extended from approximately 7 minutes to around 2 to 3 hours by simple substitution of the N-terminal Ala^2^ amino acid with Gly^2^, to effectively mask the DPP-4 binding site.^68^ In 2012, the FDA approved (Gly^2^)GLP-2, known as teduglutide, for use in people with short bowel syndrome.^63,69^ Further investigation has demonstrated that in patients with short bowel syndrome, a 5-week dosing regimen with GLP-2 markedly enhanced spinal BMD, intestinal calcium absorption rates and plasma calcium concentrations.^70,71^ In agreement with this, in postmenopausal women, GLP-2 administration results in a dose-dependent decrease in bone resorption markers.^72^ Several other studies have confirmed that GLP-2 is able to supress bone resorption both in rodents and humans.^73-75^ Indeed, clinical trials in postmenopausal women support a stimulatory impact of GLP-2 on bone growth.^75-77^ Moreover, GLP-2 has recently been shown to ameliorate age-related bone loss in mice and augment the expression of genes directly involved in bone formation.^78^

Despite this, GLP-2 receptors have only been evidenced on osteoblast-like cell lines such as MG-63 and TE-85, with identification in osteoblasts in the in vivo setting still to be fully confirmed.^79,80^ That said, the GLP-2 receptor was shown to be expressed in human osteoclast cells, confirming presence of the receptor in the setting of bone.^81^ Intriguingly, GLP-2 has also been demonstrated to be locally produced within rodent and human pancreatic islets,^82^ where it may function as an important cell-to-cell messenger and in adaptations to pancreatic islet cell stress present in obesity and diabetes. Thus, benefits of GLP-2 in bone could be linked to the gut-pancreas-bone axis.^64^ The GLP-2 receptor is clearly evident in the parathyroid gland, suggesting that effects on parathyroid hormone (PTH) secretion and/or activity may be linked to GLP-2-induced benefits on bone^73^ (Figure 2), since PTH is well recognised to be directly involved in the bone remodelling process.^83^ Indeed, consistent with this view, the antiresorptive effect of GLP-2 was shown to be abolished in patients with hypoparathyroidism.^81^ There is also a suggestion that intact gut function, especially the jejunum, is required for GLP-2 mediated benefits on bone via PTH signalling.^84^ Notably, long-acting GLP-2 receptor agonists have recently been described with effects on both rodent and human bone^85^ (Table 1), that may ultimately help fully uncover the potential of GLP-2 for the treatment of bone disorders.

### Incretins

The incretin hormones, GIP and GLP-1, play a crucial role in glucose-stimulated insulin secretion and regulation of glucose metabolism.^86^ Similar to GLP-2, both GIP and GLP-1 are GIT-derived, but also now believed to be locally produced under metabolic stress within alpha cells in pancreatic islets,^87^ as well as exerting either direct and/or indirect effects on bone health^88,89^ (Figure 2). For example, in osteoblasts, GLP-1 has been shown to promote proliferation and differentiation, enhancing their survival and inhibiting apoptosis.^88^ Additionally, GLP-1 has also been noted to inhibit osteoclast formation and activity, together helping to prevent bone loss and maintain optimal bone quality.^66,90^ In keeping with this, GLP-1 receptor KO mice present with a significant reduction in cortical bone thickness and collagen matrix maturity.^91^ Accordingly, GLP-1 receptor activation has recently been shown to improve fracture healing in a rat model of osteoporosis.^92^ However, as with GLP-2, the presence of functional GLP-1 receptors on bone remains a contentious issue,^93^ suggesting effects may be indirect and perhaps linked to its prominent insulin secretory actions (Figure 2). In support of this, GLP-1 had no effect on bone turnover in patients with T1DM.^94^

In terms of GIP effects on bone, there is conclusive evidence for the presence of GIP receptors on osteoblast and osteoclast cells^95^ (Figure 1), where GIP was revealed to reduce osteoclast activity and improve osteoblast survival of primary human bone cells.^96^ Moreover, GIP has been shown to stimulate osteoblast proliferation, differentiation and mineralisation to help promote bone formation in rodents.^97^ In keeping with this, selective KO of the GIP receptor in mice leads to reduced bone strength and quality^98,99^ In terms of related mechanisms, GIP is believed to directly modulate various signalling pathways in bone, including the cAMP/PKA pathway, the MAPK/ERK pathway and the PI3K/Akt pathway, that are crucial for bone matrix synthesis and mineralisation.^100^ There is also well-defined translation of beneficial GIP effects on bone, with GIP deceasing bone resorption in healthy volunteers.^101^ Unlike GLP-1, such effects are preserved in people with T1DM further highlighting the direct nature on the positive impact of GIP on bone.^94^ Indeed, positive effects of GIP on bone metabolism in humans are blocked by administration of a specific GIP receptor antagonist.^102^ These various observations suggest that GIT hormones, and particularly GIP which exerts positive metabolic effects directly on bone, may provide attractive therapeutic opportunities.

### Incretin analogues

As noted above, the relatively short half-life of naturally occurring peptide hormones such as GIP, GLP-1 and GLP-2 precludes clinical utilisation, leading to the requirement for enzymatically stable analogues to harness therapeutic effectiveness.^66^ In this regard, long-acting analogues of the incretin peptides, GIP and GLP-1, have been generated and examined in depth for usefulness in obesity and diabetes.^65,103^ Exendin-4 was the first GLP-1 mimetic approved for the treatment of T2DM in 2005,^104^ and studies with this peptide in an ovariectomised (OVX) rat model of bone loss revealed GLP-1 receptor mediated promotion of osteoblast activation, enhanced bone strength and partial amelioration of deterioration of trabecular microarchitecture^105^ (Table 1). In addition to this, STZ-induced diabetic OVX rats administered with the longer-acting GLP-1 mimetic, liraglutide, for 8 weeks presented with decreased osteoclast numbers, increased serum osteocalcin, protection against femoral bone mineral loss and deterioration of bone microarchitecture^106^ (Table 1). Liraglutide also exerted benefits on bone in STZ-diabetic mice, related to improvements in microarchitecture and overall bone strength^24^ (Table 1). Additionally, the GLP-1 mimetic lixisenatide reduced degradation of type II collagen and the expression of IL-6 and TNF-α in primary human chondrocytes^107^ (Table 2). Full translatability of this beneficial bone effect to the obesity-diabetes setting is provided from observations of increased bone formation in obese women treated with various clinically approved long-acting GLP-1 receptor agonists^90,108^ (Table 2), with similar findings also noted in obese men.^109^ In addition, the GLP-1 analogues exenatide and dulaglutide were shown to increase BMD and reduce fracture risk in patients with T2DM^110^ (Table 2).

Long-acting GIP analogues have also been demonstrated to exert key benefits on bone health in obesity and diabetes. For example, in STZ-diabetic mice, the well characterised enzyme resistant GIP analogue, (D-Ala^2^) GIP,^111^ augmented bone quality and effectively halted degradation of bone matrix to improve bone fracture resistance^112^ (Table 1). This finding is particularly important given that the majority of T2DM patients have normal BMD, but still present with increased bone fracture risk.^113^ This phenomenon, known as the ‘bone fragility diabetes paradox’, suggests that factors other than BMD, such as bone quality and microarchitecture, may be important in determining overall fracture risk in T2DM.^114^ To this end, the National Bone Health Alliance advises using indices of bone strength, such as changes in cortical pore and trabecular microstructure in bone, to determine osteoporosis-like disease risk in T2DM.^90^ Encouragingly, other enzymatically stabilised and long-acting GIP analogues, beyond (D-Ala^2^) GIP, have been created and characterised, including N-AcGIP.^115^ Treatment with N-AcGIP for 4 weeks improved tissue material properties of bone in normal rats, and in particular collagen maturation^116^ (Table 1). Moreover, both (D-Ala^2^) GIP and N-AcGIP reduced osteoclast formation and the extent of osteoclast resorption in vitro, an effect mediated by a reduction in intracellular calcium concentrations^21^ (Table 2). Reassuringly a similar effect of GIP on osteoclasts has also been observed in human bone cells.^96,117^

Interestingly, the dual GIP/GLP-1 receptor activating drug tirzepatide has an amino acid sequence with strong parallels to the native GIP sequence, and subsequently exhibits preference for GIP over GLP-1 receptor binding.^60^ In this regard, tirzepatide may offer an exciting new option for improving bone health in obesity and diabetes, linked to the combined direct and indirect benefits of GLP-1, and especially GIP, at the level of the skeleton.^118^ Although the precise nature of tirzepatide-receptor interactions is under debate,^119^ it is interesting to note that other dual- and triple-acting peptide molecules that tend to have GLP-1 receptor signalling as their mainstay have also been developed,^120^ with some already progressed to clinical trials.^121,122^ The overall impact of these compounds on bone in the setting of obesity and diabetes will also be of particular interest, as described below. That said, such compounds are known to induce significant weight loss, with reductions of obesity conventionally being linked to bone loss.^35^ Accordingly, bariatric surgeries that are considered gold-standard to manage obesity have been associated with increased bone fracture risk,^123^ although this finding has been questioned by others.^124^ This is interesting given that alterations in the secretion and action of gut hormones such as GIP, GLP-1 and GLP-2, are known to play a key role in the benefits of many bariatric surgeries.^125^ Thus, it is conceivable that even with weight loss induction by GLP-1 and related compounds in the clinic, direct and/or indirect benefits of these treatments on bone health could offset any potential bone loss.

### Unimolecular peptides with established effects on bone

Given recent approval of tirzepatide for T2DM,^61,118^ alongside current clinical evaluation of other similar dual- and triple-acting entities,^120^ it seems clear that the same rationale could be applied to bone-targeting unimolecular peptides. Consequently, the powerful, and likely complementary effects of GIP and GLP-2 on bone turnover,^89,126^ support this paradigm. Thus, GL-0001, a first in class GIP/GLP-2 dual-acting analogue, positively modified biomechanical parameters of bone in mice by increasing fracture resistance, preventing trabecular bone degradation and augmenting collagen cross-linking^126^ (Table 1). Moreover, GIP-GLP-2 receptor co-agonists for use in the human setting have also recently been described, that reassuringly were shown to possess similar efficacy at the level of the bone as combined treatment with respective parent peptides,^89^ suggesting translation of benefits to the clinic is conceivable. Furthermore, a peptide known as (D-Ala^2^)GIP-Oxm that co-activates GIP, GLP-1, and glucagon receptors, ameliorated reductions in bone strength following 21 days treatment in *db/db* mice^11^ (Table 1). Finally, a long-acting GLP-1/GIP/glucagon triple agonist (HM15211) protected against bone loss and significantly increased the expression of type 1 collagen-α1, -α2, and carboxylated osteocalcin in OVX rats^127^ (Table 1).

### DPP-4 inhibitors

GIP, GLP-1, GLP-2, as well as numerous other regulatory peptides, act as substrates for the ubiquitous enzyme DPP-4.^128^ Owing to the distinct benefits of GIP and GLP-1 on insulin secretion and glucose homoeostasis, drugs that inhibit DPP-4 activity are approved for the treatment of T2DM.^129^ However, unsurprisingly it has been demonstrated that DPP-4 inhibitors can lower the risk of bone fracture.^130^ Sitagliptin, a potent and selective DPP-4 inhibitor, has been shown to elevate BMD and bone quality in mice.^130^ Further investigation of the effect of sitagliptin on bone quality in diabetic rodents reveals promotion of cortical bone volume, trabecular architecture and overall bone strength.^131-133^ In high fat fed mice, once daily administration of sitagliptin for 3 weeks positively modified bone composition and improved overall bone strength and resistance to fracture.^112^ In a similar model of obesity, DPP-4 inhibition alongside metformin administration also exerted clear benefits on diabetic osteoporosis, through modulation of bone morphogenetic protein-2 (BMP-2) and sclerostin signalling.^134^ Reassuringly, in patients with T2DM, use of DPP-4 inhibitor drugs appears to be associated with favourable effects on bone health.^130^

## Potential Impact of Other Peptide Hormones and Related Drugs on Bone in Obesity and Diabetes

PTH is employed as an osteoporotic medication and established to reduce fracture risk following daily injection.^135^ Moreover, human PTH(1-34) imparts a favourable impact on bone in rodents with T2DM, linked to improved fracture healing through increased proliferation of mesenchymal stem cells.^136^ Further to this, calcitonin (CT) is a 32-amino acid hormone secreted by thyroid C cells that regulates calcium concentrations.^137^ CT is known to inhibit the resorptive activity of osteoclast cells and essentially oppose the action of PTH.^138^ In OVX rats, CT was demonstrated to promote bone formation by upregulating the expression of Wnt10b in osteoclasts,^139^ whilst CT receptor KO mice exhibit augmented bone formation.^140^ However, to date, there is a lack of clinical evidence on the overall efficacy of PTH or CT for bone disorders in the setting of obesity and diabetes. Ghrelin, a peptide that is now recognised to be synthesised within pancreatic islets as well as the stomach,^141^ is interestingly demonstrated to directly enhance osteoblast proliferation and differentiation in vitro,^142^ but further confirmation of this positive bone effect is still required. In addition, the adipocyte-derived hormone leptin, that plays a crucial role in regulating energy homoeostasis,^143^ may also be directly involved in the regulation and bone metabolism.^144^ As such, the presence of leptin receptors in adult primary osteoblasts and chondrocytes fully supports this view.^145^ Leptin may affect bone formation by activating fibroblast growth factor 23 (FGF-23), where FGF-23 is known to suppress phosphate reabsorption and vitamin D synthesis in the kidney and lately has emerged as a potential regulator of bone mineralisation.^146,147^ Leptin may also be produced locally by bone marrow adipocytes, further highlighting an important direct function on bone.^148^ Indeed, the impact of leptin on bone is thought to be independent from the action of this adipokine on energy homoeostasis.^149^ Furthermore, growth hormone (GH) is crucial for healthy bone remodelling and is known to promote osteoblast activity and bone turnover.^150^ In harmony with this, MK-677, a GH secretagogue, has been shown to elevate circulating levels of biochemical markers linked to bone production and bone resorption in humans.^151^ In women with postmenopausal osteoporosis, treatment of MK-677, either alone or in combination with a bisphosphonate, improved bone formation.^151^ However, whilst these observations are encouraging, clarification of similar benefits on bone in humans with obesity and diabetes, together with potential involvement of other peptides from the gut-bone axis, is still required. In that regard, there is emerging evidence that GIT peptides beyond GIP, GLP-1 and GLP-2, such as xenin, PYY as well as the neuroendocrine peptide vasoactive intestinal polypeptide (VIP) can also impact bone turnover,^152,153^ and these regulatory peptides merit investigation in terms of effects on bone in humans with obesity and diabetes.

## Conclusion

It is evident that obesity and diabetes significantly compromise bone health, disrupting the delicate balance between bone formation and resorption. This issue becomes even more significant in ageing populations with diminishing bone strength and given that the general diagnostic criteria for osteoporosis does not appear to be adequate for patients with obesity and diabetes. However, it is increasingly clear that drugs engineered to promote bioactivity of regulatory peptide hormones such as GIP, GLP-1 and GLP-2, can offer hope with regards to both preserving and building bone strength. These peptides have proven direct and/or indirect benefits on bone health in obesity and diabetes (Figure 2), both in the preclinical and clinical setting, although full translation of the obvious benefits in rodents is still required. Indeed, continuing advances in GIT hormone knowledge and peptide synthesis may allow for development of highly efficacious drugs that can simultaneously target multiple receptors to harness additive benefits on bone health, bringing further optimism to this field of therapeutics.

## Declarations

**Ethics approval and consent to participate:** There are no consent or ethical issues related to this review article.

**Consent for publication:** Not applicable.

**Availability of data and materials:** Data sharing is not applicable to this article because no new data were created or analysed in this study.
